# Two Different Views on the World Around Us: The World of Uniformity versus Diversity

**DOI:** 10.1371/journal.pone.0168589

**Published:** 2016-12-15

**Authors:** JaeHwan Kwon, Dhananjay Nayakankuppam

**Affiliations:** 1 Department of Marketing, Hankamer School of Business, Baylor University, Waco, TX, United States of America; 2 Department of Marketing, Tippie College of Business, University of Iowa, Iowa City, IA, United States of America; Mälardalen University, SWEDEN

## Abstract

We propose that when individuals believe in fixed traits of personality (entity theorists), they are likely to expect a world of “uniformity.” As such, they easily infer a population statistic from a small sample of data *with confidence*. In contrast, individuals who believe in malleable traits of personality (incremental theorists) are likely to presume a world of “diversity,” such that they “hesitate” to infer a population statistic from a similarly sized sample. In four laboratory experiments, we found that compared to incremental theorists, entity theorists estimated a population mean from a sample with a greater level of confidence (Studies 1a and 1b), expected more homogeneity among the entities within a population (Study 2), and perceived an extreme value to be more indicative of an outlier (Study 3). These results suggest that individuals are likely to use their implicit self-theory orientations (entity theory versus incremental theory) to see a population *in general* as a constitution either of homogeneous or heterogeneous entities.

## Introduction

Picture a little boy at an electronics store, badgering his mother to buy him a smart phone, insisting that everyone at his school has a smart phone. We know for sure that he has not researched or observed everyone; rather, he is making a naïve statistical inference from some friends that he happened to observe to the greater school population. Every day, people of all ages collect small sizes of sample data and make statistical inferences from that sample to a population. When making an inference from a sample, people ought to begin with assessing the extent to which the sample is representative of the population. They do this to be confident (to some extent) about their estimates. One might assume that an assessment of the representativeness of sample data should be typically based on mathematical or statistical reasoning, using such notions as “a smaller variation among observations is more reliable information to infer the nature of a population,” or “a larger sample size would reduce the chance of the sample mean being significantly different from the population mean.” In fact, a large body of literature shows that people are intuitively aware that larger samples are likely to yield more reliable information about a population [1,2]. Assuming that the boy was not lying to his mother, how did he then actually think about the size of his sample data and whether it was large enough to adequately represent all of the kids in his school?

We caricature two extreme versions for illustration. In support of a biased answer, in fact, numerous studies across varied domains have documented that people’s statistical inferences are often biased by non-statistical factors that are irrelevant to a population [3–5]. Likewise, it is presumable that the boy considered a small number of his friends who own smart phones as being representative of all kids in his school, just because they are close, cool, or nearby friends of his. If such were the case, his assessment about the representativeness of his sample would be affected by irrelevant characteristics. Thus, his estimate of the population mean is not likely to approach a certain level of accuracy, although he may be confident in his estimates.

Now, let’s assume that the above is not the case; instead, let’s assume that the boy inferred the population mean in a less naïve manner. He might have thought that the population should have little variation because his observations in the sample were quite consistent: “Because 6 out of 7 ‘close’ friends of his, who were ‘typical, average kids’, had smart phones (i.e., little variation in a sample), all kids in his school (i.e., the population) should have similarly little variation.” That is, he extended the sample characteristics into the nature of the population; moreover, he judged the nature of the population based on the sample characteristics at hand. Similarly, people often extend sample characteristics into a population, and utilize those when inferring a population statistic [3]. This statistical judgment process is a normative standard not only for statisticians, but also for lay people [6].

These probably strike us as two different children. Further, we might assume that the first kind is more prevalent and the second only emerges after training in statistical ideas. In this article, we question whether there is “natural” way (or default way) that lay people use in making statistical inferences, such that some people are more ready to extend sample characteristics to a population.

More specifically, we ask the following questions. Do different people hold differing naïve assumptions on the nature of a population, regardless of sample characteristics? If so, can those naïve (possibly, biased) beliefs regarding the nature of a population affect an individual’s inferences from a sample to the population? In this article, we focus on understanding people’s systematically biased beliefs about the nature of a population and their effects on the representativeness of a sample. We report four studies in which we examined whether individual differences can influence statistical inferences from a sample to a population. Specifically, we argue that an individual’s implicit self-theory orientation (originally theorized to reflect a belief in the stability or malleability of personal traits) systematically biases his/her perception regarding the nature of a population, and that these biased perceptions affect their inferences of population characteristics from sample data.

### Implicit Self-Theories

Individuals develop and use their lay theories to understand events and to interpret and predict the world around them [7]. Important for our purposes are two distinctive implicit self-theories: entity versus incremental theory. Dweck, Chiu, and Hong [8] have found that people who endorse entity theory (entity theorists) believe that their personal traits are fixed, whereas people who endorse incremental theory (incremental theorists) view their personal traits as malleable. As such, at the heart of implicit self-theory is an individual’s lay belief about the malleability of his/her own personal traits [9]. Further, one stream of research on this topic has shown that people extend their implicit self-theories to others. Erdley and Dweck [10] show that entity theorists more *easily* judge others’ dispositional traits than do incremental theorists (e.g., from a small sample of behaviors), because entity (incremental) theorists believe that not only are their personal traits fixed (malleable), but so too are other people’s traits; thus, they expect a high degree of consistency in people’s behaviors.

Other research has extended this notion to other domains. Yorkston, Nunes, and Matta [11] extend these contexts of human traits into non-human objects such as brands, suggesting that consumers transfer their implicit self-theories to brands and believe that brands hold either malleable or fixed characteristics (i.e., brand personalities). In sum, the literature suggests that compared to incremental theorists, entity theorists more easily judge the dispositional traits or the core values of what can be considered to have human-like traits, because they believe that those traits are fixed; thus, the traits can be readily inferred from a small sample of trait-related information.

### Research Questions and Conceptualization of the Studies

In this article, we attempt to expand this relatively narrow conceptualization of implicit self theory–namely, as something governing the malleability of traits associated with human or human-like objects. We propose that individuals’ implicit self-theory orientations impact their daily lives more fundamentally than what the extant research suggests. Specifically, we argue that people have the need to see aspects of the world around them either as fixed or malleable environments, so that they extend their implicit self-theory orientations to all things that do not even have human-like traits, namely everything, in order to interpret and understand the world and predict populations. How does each theorist “see” the world around him/her? Would an entity theorist see the uniqueness of each thing or person and thus, perceive the world as diverse environments (i.e., a constitution of unique entities)? Or, would an entity theorist assume that people within the same group and things within the same category are alike, such that the world is a somewhat uniform environment? In order words, if a fish in a little pond (i.e., a small size of sample data) doomed to live in a little world for its entire life imagines a great ocean outside of the pond (i.e., the intangible population), is the fish likely to assume that the great ocean be a totally different, unimaginable environment? Or that it would be the same kind of environment (but different only in size) as the little pond where it currently lives?

Extant research on the role of implicit self-theory regarding stereotype formation and application provides partial hints concerning our wider reasoning about the role of implicit self-theory. For example, Levy, Stroessner, and Dweck [12] found that compared to incremental theorists, entity theorists make more stereotypical trait judgments of ethnic and occupational groups, presumably because entity theorists believe there are smaller trait differences between the group members than incremental theorists do. Although they provide preliminary evidence that entity theorists may perceive greater group homogeneity in social judgments than incremental theorists, these findings are still questionable in supporting our theory. Because extant research on stereotyping has solely focused on groups of people with trait-related information, still unknown are people’s tendencies to understand the world at large, not limited to a social judgment, as either invariant or dynamic based on their implicit self-theory orientations. However, at an overall level, this stream of research suggests the possibility that entity theorists view many aspects as unchanging, and that their belief about their personal traits not changing is just one other aspect of this overall belief structure. As discussed, we propose that people’s implicit self-theory orientations have a more profound impact on their perceptions of the world and on their daily judgments and decisions, than on the context in which the current notion confines the effect (i.e., things that hypothetically have human-like traits).

This reasoning led us to formulate the prediction that entity theorists will estimate the nature of a population from a small set of sample data with a greater level of confidence than will incremental theorists. Note that although built upon the extant stereotype research, the current research differs in that our predictions are not limited to the trait judgments of a group of people. Rather, we expect the same effect on the judgments on both social and non-social targets. Note that the extant stereotype research suggests the extension of one’s implicit self-theory orientation to a social target (for e.g., “if my personality if fixed, other people’s personalities are fixed as well”) is the underlying mechanism for the phenomenon–compared to incremental theorists, entity theorists make more stereotypical trait judgments of groups of people. Thus, this stream of research does *not* predict that compared to incremental theorists, entity theorists are more likely to be confident in making statistical inferences on “*non-social”* populations. For example, let’s say one is given a small sample of the consumer review scores on a certain product and asked to estimate the mean of the all of the consumer review scores (i.e., population mean) on the product. The extension of implicit self-theory account would not predict any difference in the estimates between the theorists.

In the following sections, we report four studies to establish and provide evidence of this effect–entity theorists are more likely to fall victim to statistical sampling biases than incremental theorists are. In particular, Study 1a and 1b test the divergent extent of “confidence” between entity and incremental theorists when estimating the population mean from a sample mean. Study 2 directly examines the extent to which each theorist expects representativeness of sample data. Study 3 delves more deeply into the representativeness of a sample by examining the effect of an extreme value in a sample dataset when estimating a population mean. A participant’s implicit self-theory orientation was manipulated in all of the four studies, such that the results of each study can be considered as having originated from the causal effect of implicit self-theory.

## Study 1a: Estimating the Population Mean from Sample Data with a Different Number of Observations

### Sample and Procedure

One hundred-fifty undergraduate students in a large public university in the U.S. were recruited and randomly assigned to a 2 (implicit self-theory: entity vs. incremental theory) X 3 (number of observations: 2 vs. 4 vs. 6) between-subjects design (male = 68.21%, average age = 20.9). All procedures were approved by the Hawk IRB at the University of Iowa (IRB ID #: 201012735). After s/he read the written consent document and agreed on his/her participation to this research, each participant was first given one of the two mock scientific articles presenting views consistent with entity theory or incremental theory [13]. To ensure that the articles successfully primed the appropriate implicit theories, participants completed the eight-item implicit self-theory measure scale [12]. A one-way ANOVA on the combined implicit self-theory scale (alpha = .91), with higher scores indicating a stronger belief in entity theory, yielded a significant main effect of the manipulation (*M*_Ent_ = 4.59 vs. *M*_Inc_ = 2.87, *F*(1,149) = 142.39, *p* < .001, *η*^2^ = .49).

Next, for a cover story, participants were told that Mrs. Sarah Arter, an alumna of their college, has donated to a charity for 15 years. They were then given one of the three partial records of her monetary contributions each year: the records of the most recent 2 years ($535 and $715), or those of the most recent 4 years ($535, $715, $680 and $570), or those of the most recent 6 years ($535, $715, $680, $570, $595 and $695). Note that the range ($535.^00^ to $715.^00^) and the average ($625.^00^) of the samples remain the same across the three conditions. Participants were then asked to estimate the possible minimum and maximum average annual monetary contributions from Mrs. Arter (e.g., “What could be the range that the real average annual monetary contribution from Mrs. Arter would fall in between? Somewhere between _____ and ____”). Finally, the participants were fully debriefed, thanked and dismissed.

### Results and Discussion

**Fig 1:**
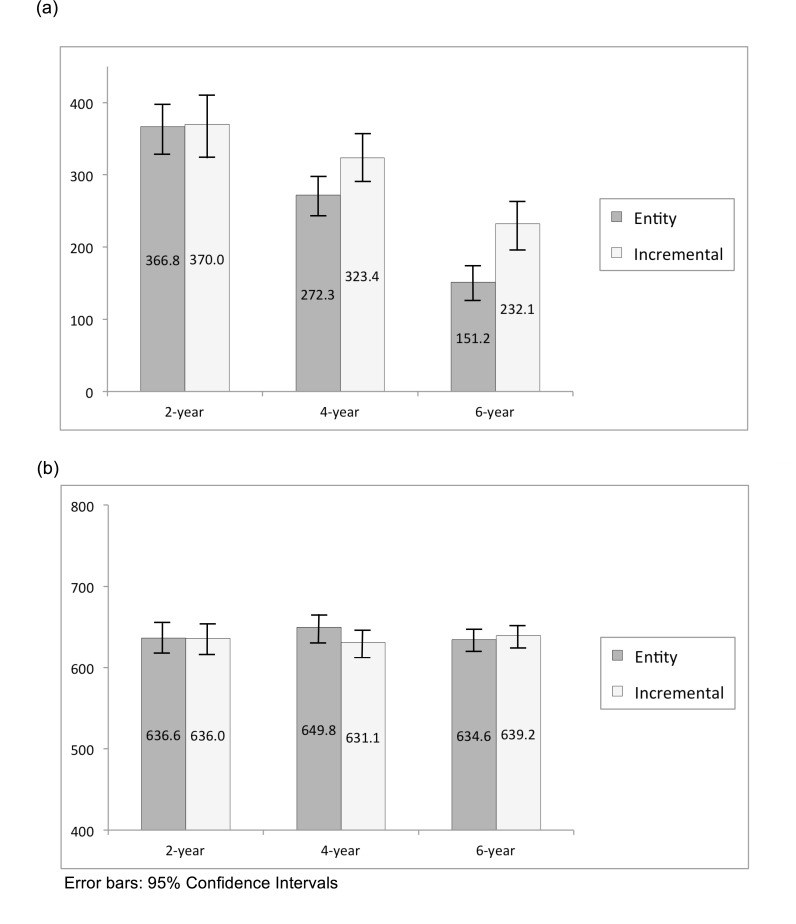
Study 1a –Estimated Average of the Monetary Contribution per Year. **(a) The range of the estimated average (the difference between the possible maximum and the minimum averages (unit: US dollar). (b) The midpoint of the range (unit: US dollar).** Error bars: 95% Confidence Intervals.

A 2 X 3 ANOVA on the range of the estimates (the difference between the max. and the min.) yielded a significant interaction effect (*F*(2,149) = 3.49, *p* = .033, *η*^2^ = .05), along with the main effects of implicit self-theory (*F*(1,149) = 13.95, *p* < .001, *η*^2^ = .09) and of the number of observations in a sample dataset (*F*(2,149) = 71.72, *p* < .001, *η*^2^ = .50). Planned comparisons revealed that compared to incremental-primed participants, entity-primed participants estimated the average with an equal or smaller range across all of the number of observation conditions. This effect was especially true when given the 4-year (*F*(1,50) = 8.39, *p* = .001, *η*^2^ = .15) or 6-year dataset (*F*(1,48) = 17.90, *p* < .001, *η*^2^ = .28). A 2 X 3 ANOVA on the midpoint of the range, however, did not yield any significant effect; the interaction effect did not approach significance (*F*(2,149) = 2.08, *NS*), nor did the main effects (*F*(1,149) = 1.00, *NS*, *F*(2,149) = .28, *NS*) (Fig 1). In order words, while both incremental and entity theorists estimated the same mean level of contribution, entity theorists were quicker to infer lower variance–as the sample size increased, entity theorists were quick to restrict the range while incremental theorists were more reluctant to do so.

Recall our early discussion that we expect this to be true no matter whether the nature of the population is social or non-social. One could argue that charitable contributions are somewhat social in nature. Thus, in the next study, we use a non-social target and attempt to replicate the results of Study 1a.

## Study 1b: Estimating the Population Mean for a Non-Social Target

Participants were given a small sample of the consumer ratings on a fictitious 3D-TV and asked to estimate the mean of all of the consumer ratings (i.e., population mean) on the product.

### Sample and Procedure

One hundred and thirty-eight undergraduate students were randomly assigned to a 2 (implicit self-theory: entity vs. incremental theory) X 3 (number of observations: 2 vs. 4 vs. 6) between-subjects design (male = 61.59%, average age = 20.8). Upon arrival, each participant’s implicit theory orientation was primed either to entity or incremental theory using the same procedures used in Study 1a.

Next, participants were given a photocopy of a fictitious 3D-TV and told that there were a total of 20 consumer ratings for the 3D-TV in a consumer review website. They were then given one of the three samples of the consumer ratings: 3 ratings (3, 4, and 5 stars out of 5 stars), 5 ratings (3, 3, 4, 5, and 5 stars), or 7 ratings (3, 3, 4, 4, 4, 5, and 5 stars) (S1 Fig). Note that the range and the average of the samples remain the same across the three conditions. Participants were then asked to estimate the possible minimum and maximum average ratings of the total of twenty consumer ratings on the website.

Next, to ensure that the articles successfully primed the appropriate implicit theories, participants completed the implicit self-theory scale used in Study 1a. A one-way ANOVA on the combined implicit self-theory scale (alpha = .89) yielded a significant main effect of the manipulation (*M*_Ent_ = 4.60 vs. *M*_Inc_ = 3.35, *F*(1,136) = 57.77, *p* < .001, *η*^2^ = .30).

### Results and Discussion

A 2 X 3 ANOVA on the range of the mean estimates (the difference between the max. and the min.) yielded a marginally significant interaction (*F*(2,132) = 2.57, *p* = .081, *η*^2^ = .04), along with the significant main effects of implicit self-theory (*F*(1,132) = 9.98, *p* < .01, *η*^2^ = .07) and of the number of observations in a sample dataset (*F*(2,132) = 17.64, *p* < .001, *η*^2^ = .21). A 2 X 3 ANOVA on the midpoint of the range, however, did not yield any significant effect; the interaction effect did not approach significance (*F*(2,132) = 1.31, *NS*), nor did the main effects (*F*(1,132) = 2.68, *NS*, *F*(2,132) = 1.10, *NS*). This pattern of results thus replicates those found in Study 1a.

In Study 1b, we used a non-social stimulus and replicated the results of Study 1a. Studies 1a and 1b together provide an initial piece of evidence that each theorist is likely to hold different expectations regarding the nature of a population in general. We delve more into this idea in the following two studies.

## Study 2: Estimating Population Size from a Population Mean

### Sample and Procedure

One hundred and nineteen participants at the same university were randomly assigned to a 2 (implicit self-theory: entity vs. incremental theory) X 2 (population mean given: in-range of sample dataset– 81.5 vs. out-range– 86.5) between-subject design (male = 57.98%, average age = 21.3). Upon arrival, the participants’ implicit self-theory orientations were primed with either entity or incremental theory using the same procedure as in Study 1.

As a cover story, they were told that a class in a high school nearby had recently taken an aptitude test (total score = 100) and that among the class, the test scores of only 5 students were randomly selected to be open to the public, which were, respectively, 77, 80, 81, 82, and 85. They were also told that the mean of these five observations was 81.0 (i.e., sample mean), and they ranged from 77 to 85 (i.e., sample range). Along with the five test scores, participants under the in-range condition were told that the average of the class (i.e., population mean) was 81.5, and that participants under the out-range condition were told that the average was 86.5. They were both asked to guess the total number of students in the class. The purpose of Study 2 is to examine the extent to which each theorist expects representativeness of sample data. We expect that there will be no difference in their estimated number of students between the theorists for the in-range condition, but the difference will emerge in the out-range conditions.

### Results and Discussion

A 2 X 2 ANOVA on the estimate of the number of students in the class was conducted. Significant main effects of implicit self-theory (*M*_Ent_ = 37.31 vs. *M*_Inc_ = 33.02, *F*(1,118) = 4.59, *p* = .034, *η*^2^ = .04) and of the given average score (*M*_In-range_ = 23.39 vs. *M*_Out-range_ = 47.50, *F*(1,118) = 150.48, *p* < .001, *η*^2^ = .57) emerged. These main effects were qualified by the interaction term (*F*(1,118) = 5.77, *p* = .018, *η*^2^ = .05). A decomposition of this interaction revealed that entity theorists’ estimations regarding the number of students were greater than those of incremental theorists only for the out-range condition (*M*_Ent_ = 51.97 vs. *M*_Inc_ = 43.03, *F*(1,57) = 5.56, *p* = .022, *η*^2^ = .09); the difference between the theorists for the in-range condition did not approach significance (*M*_Ent_ = 23.13 vs. *M*_Inc_ = 23.65, *F*(1,60) = .15, *NS*).

When the population mean given was in the range of the observations within the sample data (i.e., in-range condition), participants did not need to consider any other factors upon inferring the population size. In this situation, the estimates of population size by entity theorists were not different from those by incremental theorists. This suggests that both theorists had similar reference points in terms of the number of students in a high school class, and that they both were similar in making statistical inferences from a sample to a population (consistent with the results of the mid-point of the range in Study 1). When participants were given the out-range population mean (i.e., out-range condition), however, they may have considered either of the following two possibilities: 1) the population may have consisted of a relatively *larger number* of students who did *a little better* than the five students in the sample data; or 2) the population may have consisted of a relatively *small number* of students, some of who did *much better* than the students in the sample. The former possibility assumes that the sample data were still somewhat representative of the population, and that the population, including the observations in the sample, may have consisted of more *homogeneous* entities. In contrast, the latter possibility assumes that the sample data were not representative of the population, and that the population may have consisted of *heterogeneous* entities with much higher variance in their test scores. Consequently, the results under the out-range condition in this experiment indicate that entity theorists still expect a certain level of representativeness in the sample data and a homogeneous population, whereas incremental theorists consider the possibility that the sample may not be representative of the population, and that the population may be more diverse.

## Study 3: Differing Perceptions toward an Extreme Value in Sample Data when Estimating a Population Mean

In Study 3, we delve more deeply into the representativeness of a sample by examining the effect of an extreme value in a sample dataset when estimating a population mean, rather than a population size. We expect that when estimating a population mean, entity theorists may perceive an extreme value in the sample as being more likely to be an outlier, while incremental theorists may perceive it as being more likely to represent a systematic difference in the population.

### Sample and Procedure

Two hundred twenty-one participants at the same university were randomly assigned to a 2 (implicit self-theory: entity vs. incremental theory) X 2 (extreme value: with an extreme value vs. without an extreme value) X 3 (standard deviation: 1 SD vs. 2 SD. vs. 3 SD) between-subject design (male = 62.44%, average age = 21.2). After being primed with either implicit self-theory, participants were given a dataset (along with the sample mean and the SD information), depending on their extreme-value and SD conditions (see Table 1). As a cover story, they were told that forty-eight students in a nearby high school had recently taken an aptitude test, and the dataset given included only nine (or ten) out of forty-eight scores, which had been randomly selected to be publicized. Participants were asked to estimate the average of the forty-eight students (i.e., population mean).

**Table 1:** The *Sample* Datasets Given to Participants by Experimental Condition.

Cond.	Implicit Self-Theory	Extreme Value	SD	Dataset	Avg.
1	Entity	*Without* an extreme value	1.0	{80, 80, 81, 79, 81, 81, 80, 78, 80}	80.0
2			2.0	{82, 80, 81, 78, 81, 80, 78, 77, 83}	80.0
3			3.0	{84, 76, 82, 77, 83, 81, 77, 78, 82}	80.0
4		*With* an extreme value	1.0	{80, 80, 81, 79, 81, 81, 80, 78, 80, ***90***}	81.0
5			2.0	{82, 80, 81, 78, 81, 80, 78, 77, 83, ***90***}	81.0
6			3.0	{84, 76, 82, 77, 83, 81, 77, 78, 82, ***90***}	81.0
7	Incremental	*Without* an extreme value	1.0	{80, 80, 81, 79, 81, 81, 80, 78, 80}	80.0
8			2.0	{82, 80, 81, 78, 81, 80, 78, 77, 83}	80.0
9			3.0	{84, 76, 82, 77, 83, 81, 77, 78, 82}	80.0
10		*With* an extreme value	1.0	{80, 80, 81, 79, 81, 81, 80, 78, 80, ***90***}	81.0
11			2.0	{82, 80, 81, 78, 81, 80, 78, 77, 83, ***90***}	81.0
12			3.0	{84, 76, 82, 77, 83, 81, 77, 78, 82, ***90***}	81.0

NOTE: The sample mean of every without-an-extreme-value condition remained the same (80.0). With-an-extreme-value conditions added a score of 90 to the datasets of corresponding without-an-extreme-value conditions. As a result, the SDs in the with-an-extreme-value conditions are slightly higher than those of the corresponding conditions.

### Results and Discussion

**Fig 2:**
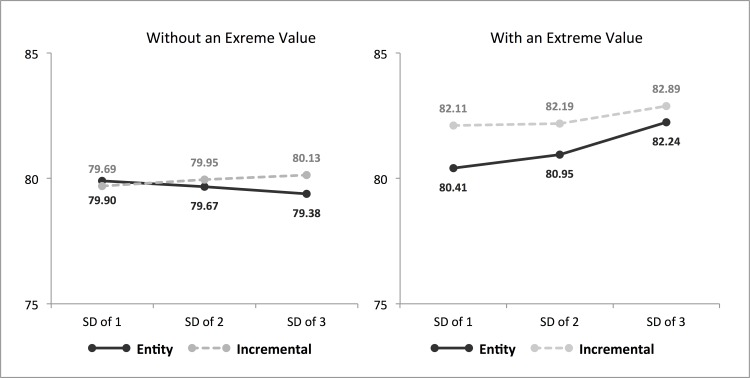
Study 3 –Estimated Average of the Class.

The two-way extreme value X standard deviation interaction emerged (*F*(2,220) = 7.08, *p* = .001, *η*^2^ = .06). However, planned comparisons revealed that the effects of the standard deviation were significant only at the with-an-extreme-value condition (*M*_1SD_ = 81.31, *M*_2SD_ = 81.55, *M*_3SD_ = 82.54, *F*(1,111) = 22.92, *p* < .001, *η*^2^ = .30), but not at the without-an-extreme-value condition (*M*_1SD_ = 79.80, *M*_2SD_ = 79.81, *M*_3SD_ = 79.79, *F*(1,108) = .02, *NS*) (Fig 2).

More importantly, the two-way implicit self-theory X extreme value interaction emerged as well (*F*(1,220) = 8.95, *p* = .003, *η*^2^ = .04), along with the main effects of implicit self-theory (*F*(1,220) = 22.81, *p* < .001, *η*^2^ = .10). Planned comparisons revealed that both theorists estimated higher population means when the sample datasets included an extreme value, than when the sample did not have an extreme value (*F*(1,220) = 170.54, *p* < .001, *η*^2^ = .45). The magnitude of this extreme-value effect, however, was greater among incremental theorists (*M*_With-ex_ = 82.39, *M*_Without-ex_ = 79.93, *F*(1,111) = 209.45, *p* < .001, *η*^2^ = .66) than among entity theorists (*M*_With-ex_ = 81.26, *M*_Without-ex_ = 79.66, *F*(1,108) = 35.98, *p* < .001, *η*^2^ = .26).

These effects were qualified by a three-way interaction (*F*(2,220) = 3.50, *p* = .032, *η*^2^ = .03) (Fig 2). A decomposition of this interaction revealed that when the sample data did not include an extreme value, the estimated population means were similar between entity and incremental theorists across all standard deviation conditions (all *p* > .156). However, when the sample data included an extreme value, incremental theorists estimated a higher population mean than entity theorists across all standard deviation conditions. As predicted, the magnitude of this difference between entity and incremental theorists became smaller as the SD of the sample data increased: under 1SD (*F*(1,35) = 36.60, *p* < .001, *η*^2^ = .52); under 2SD (*F*(1,36) = 15.01, *p* < .001, *η*^2^ = .30); and under 3SD (*F*(1,38) = 6.44, *p* = .016, *η*^2^ = .15). These results suggest that entity theorists were more likely to believe in a homogeneous population, such that they considered the extreme value in the sample dataset to be “an outlier”–most students would be closer to the sample average. In contrast, incremental theorists were more likely to believe in a heterogeneous population, such that they considered the extreme value to be indicative of “a systematic difference” among students in the population. Said differently, entity theorists appeared to think the sample without the outlier was more representative of the population and the outlier was a ‘special case’, unrepresentative of the population, while incremental theorists were reluctant to treat the out-of-range value as an ‘outlier,’ ‘special,’ or ‘unrepresentative’.

## Conclusion

A well-established notion is that individuals transfer their implicit self-theories to others [10] and even to non-human objects that could have hypothetically human-like traits, such as brands or products [11]. We show that individuals’ implicit self-theory orientations also influence their perceptions toward things with non-human-like traits, groups of things, and presumably the world around them. In this article, we showed that individuals were likely to use their implicit self-theories to see a population as a constitution either of homogeneous or heterogeneous entities. Consequently, compared to incremental theorists, entity theorists estimate a population mean from a sample with a greater level of confidence (i.e. underestimate the confidence interval) (Study 1a and 1b), expect more homogeneity among entities in the general population (Study 2), and perceive an extreme value as being more likely to represent an outlier (Study 3). In short, the current research is the first to document that compared to incremental theorists, entity theorists are more likely to fall victim to statistical sampling biases. What is unexplored yet in the literature is whether entity theorists are more likely to fall for the base rate fallacy [14] and/or are more likely to fall for the availability heuristic bias [15] than incremental theorists are. In the future research, it would be a fruitful and interesting next step to research on those topics and provide a more holistic view on the influence of an individual’s implicit self-theory orientation on his/her statistical judgments.

As discussed earlier, extant research on stereotype has shown that compared to incremental theorists, entity theorists make more stereotypical trait judgments about a group of people [12]. Note that, however, this stream of research has solely focused on groups of people with trait-related information, such that still unknown were people’s tendencies to understand the world (i.e., the general populations) as either invariant or dynamic based on their implicit self-theory orientations. The findings of the current research suggest that an individual’s implicit self-theory orientation has a more fundamental impact on his/her judgments than what the extant literature suggests.

What process, then, accounts for these differing perceptions regarding a population between each type of theorist? We propose that people have a need to see aspects of the world around them either as fixed or as malleable environments to interpret and understand the world, to predict populations, and to make daily judgments. We argue that one of the simplest and most reliable ways for people to do so is to extend their lay beliefs about the malleability of their own personal traits (their implicit self-theory orientations) to everything around them in the world–in this sense, we *see* our own reflection in the world around us. This research provides preliminary support for this view, especially with regard to the influence of individuals’ implicit self-theory orientations to their perceptions of populations. However, it would be premature to conclude that people solely use their implicit self-theories to infer the nature of populations and to understand the world. It is presumable that other unidentified factors play more direct roles in inferring the nature of a population. For example, people may be able to quickly develop another lay theory about the malleability of specific target populations (e.g., the generosity of their alumna in our Study 1) when primed with either implicit self-theory orientation. Note that the purpose of this research is to open up a potentially new spectrum for understanding the influence of implicit self-theory on our lives.

The implicit self-theory literature has documented that individuals are chronically predisposed to one or the other type of implicit self-theories [16]. That is, an individual’s implicit self-theory orientation can be conceived as a stable individual difference. Relatively recently, however, a growing body of research has shown that individuals can hold both implicit self-theories simultaneously [9,12,13,16,17]. Although one theory may be dominant, the other one remains available and can become accessible under specific situational circumstances [11]. Therefore, an individual’s implicit self-theory orientation can be situationally primed as we temporarily activated either of the implicit self-theory orientations among participants in the current research.

The findings of the current research suggest that the fish in a little pond could perceive the great ocean in either way: if the fish was an incremental theorist, it would have assumed that the great ocean could be a totally different, unimaginable environment; if it was an entity theorist, however, it would have posited that the ocean was just another pond, only larger in scale.

## Supporting Information

S1 Fig An Example of the Stimuli (the 7-ratings conditions). (TIF)

S1 File Data. (XLSX)

## References

[1] Chesney DL, Obrecht NA. Statistical judgments are influenced by the implied likelihood that samples represent the same population. Mem Cogntion. 2012 4 1;40(3):420–33. 10.3758/s13421-011-0155-3 22033973

[2] Obrecht NA, Chapman GB, Gelman R. Intuitive t-tests: Lay use of statistical information. Psychonomic Bulletin Rev. 2007 12 1;14(6):1147–52. 10.3758/bf03193104 18229488

[3] Kahneman D, Tversky A. Subjective probability: A judgment of representativeness. Cognitive Psychol. 1972 7 31;3(3):430–54.

[4] Mishra A, Mishra H, Nayakankuppam D. The group-contagion effect: The influence of spatial groupings on perceived contagion and preferences. Psychol Sci. 2009 7 1;20(7):867–70. doi: 10.1111/j.1467-9280.2009.02371.x 19493323 10.1111/j.1467-9280.2009.02371.x

[5] Windschitl PD. Judging the accuracy of a likelihood judgment: The case of smoking risk. J Behav Decis Making. 2002 1 1;15(1):19–35.

[6] Kushnir T, Xu F, Wellman HM. Young children use statistical sampling to infer the preferences of other people. Psychol Sci. 2010 8 1:21(8):1134–40. doi: 10.1177/0956797610376652 20622142 10.1177/0956797610376652 PMC3785083

[7] Lickel B, Hamilton DL, Sherman SJ. Elements of a lay theory of groups: Types of groups, relational styles, and the perception of group entitativity. Pers Soc Psychol Rev. 2001 5 1;5(2):129–40.

[8] Dweck CS, Chiu C, Hong Y. Implicit theories and their role in judgments and reactions: A world from two perspectives. Psychol Inq. 1995 10 1;6(4):267–85.

[9] Kwon J, Nayakankuppam D. Strength without elaboration: The role of implicit self-theories in forming and accessing attitudes. J Consum Res. 2015 4 29:42(2):316–39.

[10] Erdley CS, Dweck CS. Children’s implicit theories as predictors of their social judgments. Children Development, Child Dev. 1993 6 1;64(3):863–78. 8339700

[11] Yorkston EA, Nunes JC, Matta S. The malleable brand: The role of implicit theories in evaluating brand extensions. J Marketing. 2010 1 1;74(1):80–93.

[12] Levy SR, Stroessner SJ, Dweck CS. Stereotype formation and endorsement: The role of implicit theories. J Pers Soc Psychol. 1998 6;74(6):1421–36.

[13] Chiu C, Hong Y, Dweck CS. Lay dispositionism and implicit theories of personality. J Pers Soc Psychol. 1997 7;73(1):19 9216077 10.1037//0022-3514.73.1.19

[14] Bar-Hillel M. The base-rate fallacy in probability judgments. Acta Psych. 1980 5 31:44(3):211–33.

[15] Tversky A, Kahneman D. Availability: A heuristic for judging frequency and probability. Cognitive Psych. 1973 9 30:5(2):207–32.

[16] Kwon J, Seo Y, Ko D. Effective luxury-brand advertising: The ES–IF matching (Entity–Symbolic versus Incremental–Functional) model. J Advert. In press.

[17] Park JK, John DR. Got to get you into my life: Do brands personalities rub off on consumers? J Consum Res. 2011:37(4):655–69.

