# Supplementary figures and images for: Two Different Views on the World Around Us: The World of Uniformity versus Diversity
Source: PLoS One. 2016 Dec 15;11(12):e0168589. doi: 10.1371/journal.pone.0168589 (PMC5158088; doi:10.1371/journal.pone.0168589)

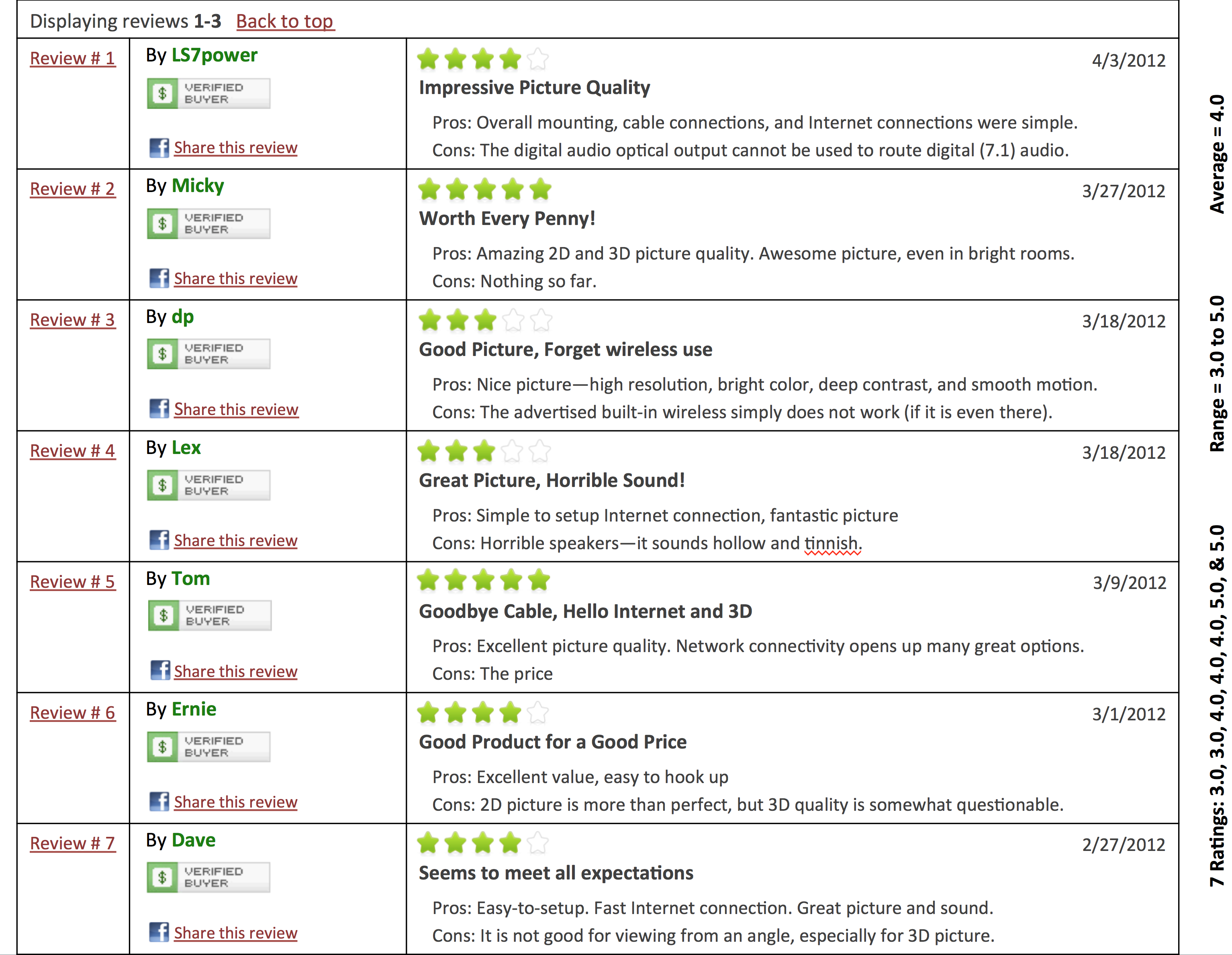

Supplement: S1 Fig — (TIF) [file pone.0168589.s001.tif]
